# Supplementary material for: Nanoimaging of resonating hyperbolic polaritons in linear boron nitride antennas
Source: Nat Commun. 2017 Jun 7;8:15624. doi: 10.1038/ncomms15624 (PMC5467227; doi:10.1038/ncomms15624)
Supplement: Supplementary Information — Supplementary Figures [file ncomms15624-s1.pdf]

(a)

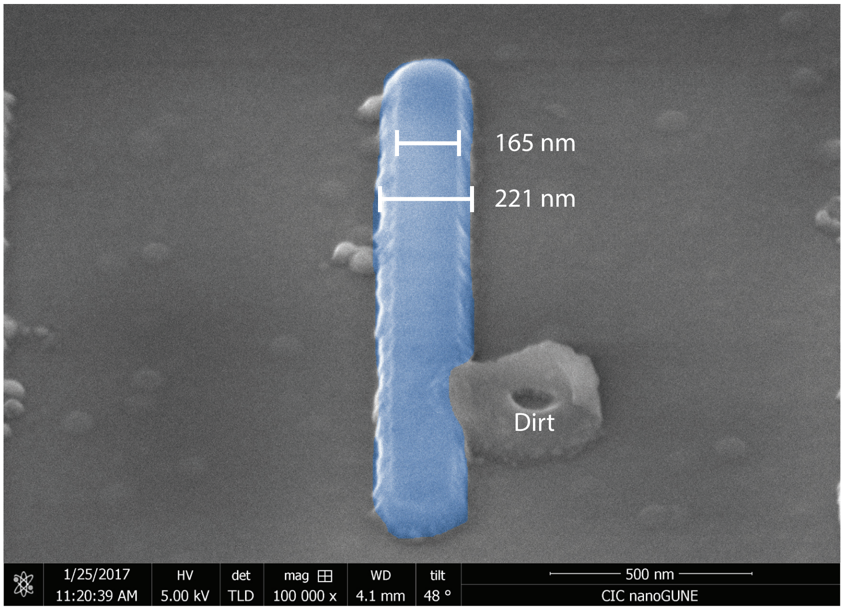

(b)

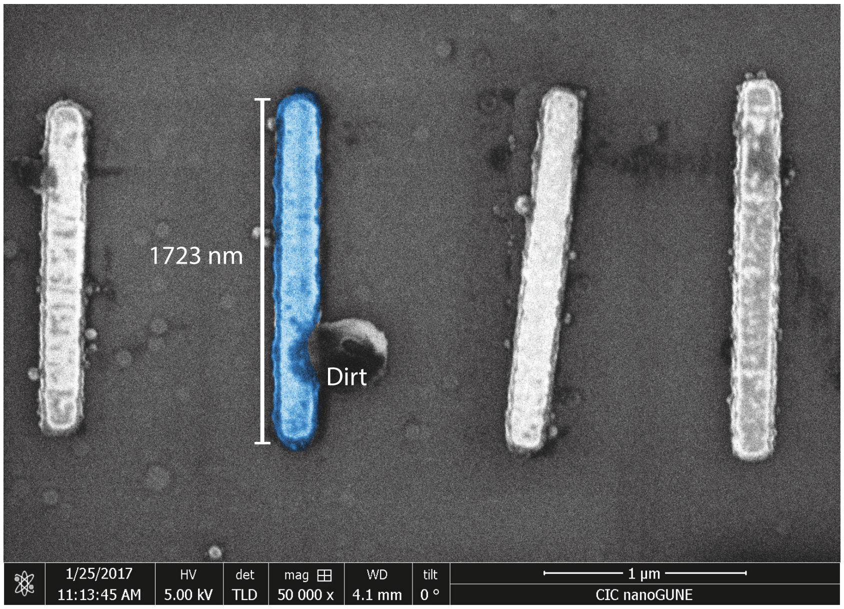

Supplementary Figure 1: Scanning electron microscope (SEM) image of the antenna analyzed in Figures 2 and 3. (a) SEM image with the sample tilted by 48º relative to the surface normal, in order to better recognize its transverse cross-section. The h-BN antenna is colored blue. Note that the ring-like particle next to the antenna was deposited after the results of Figures 2 and 3 were obtained, probably by applying too much laser power that led to melting of the platinum coating of the tip. (b) Top-view SEM image of the antenna shown in panel (a).

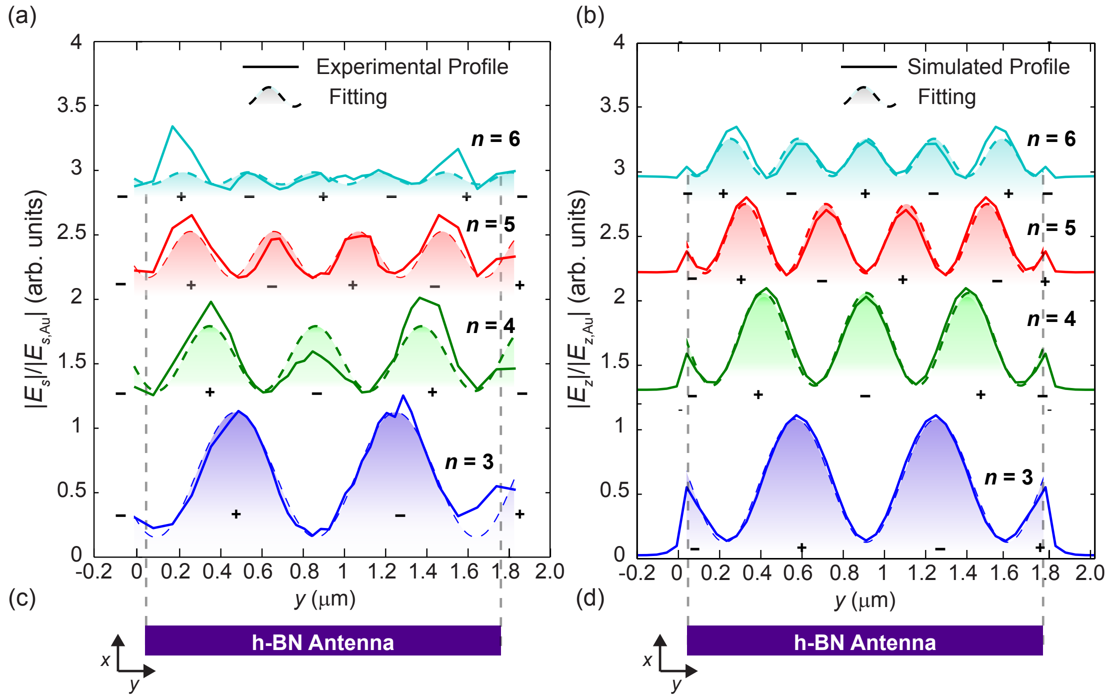

Supplementary Figure 2: Determination of the wavevector of the SM0-S modes from the spectral line scans of Figures 2d and e. (a) Solid lines: Experimental near-field amplitude profiles $|E_s|/|E_{s,Au}|$ at the resonance frequencies ω_n with n = 3 to 6 (extracted from Figure 2d). Note that the line profiles have a length of 1810 nm. The positions y = 0 nm and y = 1810 nm mark the two very extremities of the antenna in the topography image shown in Figure 2c. Dashed lines: Fittings of the experimental profiles with the function $A_{n,0} + A_{n,1}\sin(2 \cdot q_n \cdot y + \alpha_n)$, where q_n is the momentum of the waveguide mode. α_n, $A_{n,0}$, and $A_{n,1}$ are fitting parameters, which are not further considered. The plus and minus signs represent the direction of the vertical component of the electric field. (b) Simulated (solid lines) and fitted (dashed lines) near-field profiles $|E_z|/|E_{z,Au}|$, analogous to panel (a). The antenna length in the simulations was 1723 nm, corresponding to the experimental antenna length that was determined in the Supplementary Figure 1. (c,d) Sketch of the antenna with a length of 1723 nm, which was measured in the Supplementary Figure 1.

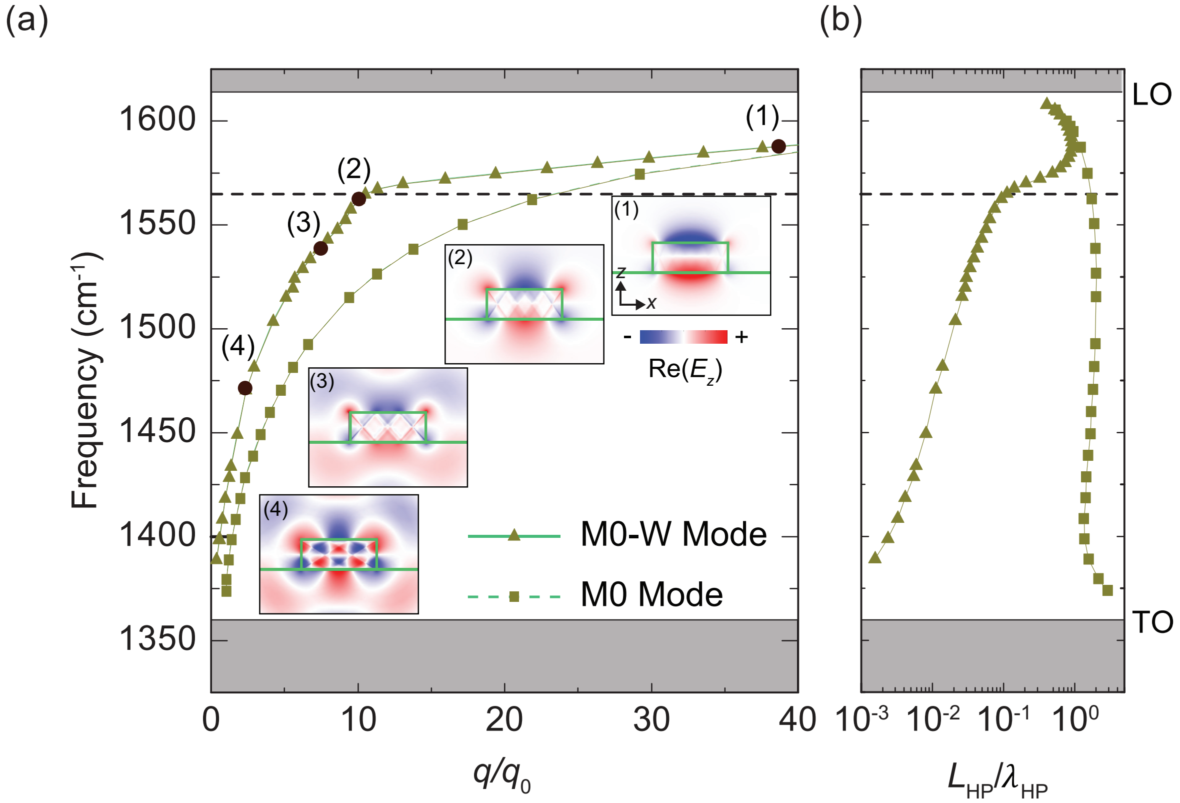

Supplementary Figure 3: Calculated dispersion and relative propagation length of the M0 and M0-W modes. The width of the rectangular waveguide is 190 nm. The height of both the waveguide and the infinite slab is 74 nm. (a) Dispersion of the M0-W mode in the rectangular waveguide (square green symbols, the dashed green line is a guide to the eye) and of the M0 mode in an infinite slab (triangular green symbols, the solid green line is a guide to the eye). The insets show snapshots of the vertical component of the electric field at the frequencies marked by black dots. They show that the number of nodes of the electric field on the waveguide surfaces are constant all along the green solid curve (two nodes in the horizontal direction and one node in the vertical direction), thus verifying that the solid curve reveals the dispersion of one mode. (b) Relative propagation length of the M0 (dashed green line) and M0-W (solid green line) modes, L_{HP}/λ_{HP}, where λ_{HP} is the wavelength and L_{HP} the propagation length. We observe a strong reduction of the relative propagation length of the M0-W mode at a frequency (marked by dashed black horizontal line) where the dispersion curve abruptly bends. For a better understanding of this behavior, a more detailed analysis is needed, which, however, would go far beyond the scope of the present work.

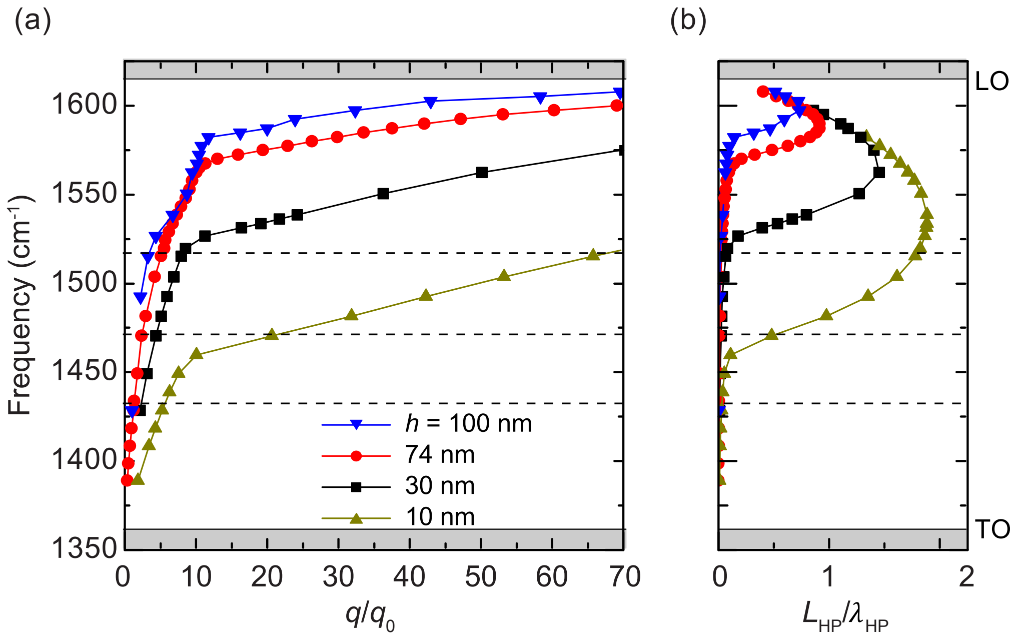

Supplementary Figure 4: Dispersion (a) and relative propagation length (b) of the M0-W mode for different waveguide heights *h*. The width of the rectangular waveguide is 190 nm. The symbols show the calculated points of the dispersion. The solid lines are guides to the eye. The dashed black lines indicate the frequencies at which we study in the Supplementary Figure 5 the relative propagation length of the M0-W as a function of waveguide height and width. Note that the frequency where the dispersion curve abruptly bends (that is, where the relative propagation length abruptly increases with increasing frequency) is decreasing when the waveguide height *h* decreases. As a consequence, the relative propagation length at a fixed frequency increases when the waveguide height is reduced.

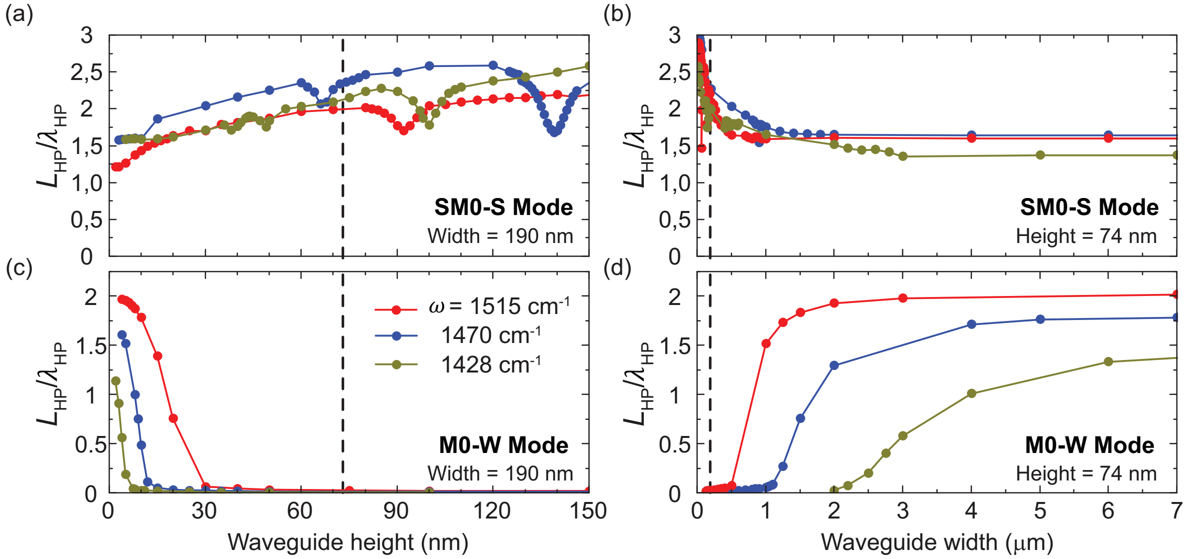

Supplementary Figure 5: Calculated relative propagation length of the SM0-S and M0-W modes. (a) Relative propagation length λ_{HP}/L_{HP} of the SM0-S mode as a function of the waveguide height for three different frequencies. λ_{HP} is the wavelength and L_{HP} is the propagation length. The width of the waveguide is 190 nm. The vertical dashed black line marks the height of the antenna studied in Figure 2. (b) Relative propagation length of the SM0-S mode as a function of waveguide width for three different frequencies. The height of the waveguide is 74 nm. The vertical dashed line indicates the width of the antenna studied in Figure 2. (c,d) Same analysis as in panels (a) and (b) but for the M0-W mode. In all calculations the SiO_2 substrate has been taken into account. The calculations show that the relative propagation length of the SM0-S mode only slightly decreases with decreasing height of the waveguide (panel a), while a significant increase by nearly a factor of two is observed for decreasing the waveguide width below 1 μm (panel b). For the M0-W mode a quite different trend is found. The relative propagation length is negligible small for large waveguide heights, however, it increases substantially when the height is decreased below 30 nm (panel c), which can understood with the help of Supplementary Figures 3 and 4. We further find that the relative propagation length decreases with decreasing waveguide width. It seems that rectangular waveguides with a large aspect ratio width/height are required to support M0-W modes with propagation lengths similar to that of the SM0-S mode. The longest propagation lengths, interestingly, are found for the SM0-S mode when the aspect ratio height/width is large. Although more detailed and systematic studies are needed to fully understand the properties of these waveguide modes, the results clearly show that a variety of h-BN waveguides with sub-100 nm size width and/or height can support propagating hyperbolic phonon polariton modes at mid-infrared frequencies, which could lead to extremely miniaturized antennas and circuits for sensing and thermal transport applications.
